# Generation of a conditional transgenic mouse model expressing human Phospholipase A2 Receptor 1

**DOI:** 10.1038/s41598-020-64863-y

**Published:** 2020-05-18

**Authors:** Sara Jaber, Delphine Goehrig, Philippe Bertolino, Amélie Massemin, Franck Bihl, Joëlle Chabry, Gérard Lambeau, David Vindrieux, David Bernard

**Affiliations:** 1 Inserm U1052, CNRS UMR 5286, Université de Lyon & Centre Léon Bérard, Centre de Recherche en Cancérologie de Lyon, Lyon, France; 2 Université Côte d’Azur (UCA), Centre National de la Recherche Scientifique (CNRS), Institut de Pharmacologie Moléculaire et Cellulaire (IPMC), UMR7275 Valbonne, Sophia Antipolis France

**Keywords:** Biological models, Medical research

## Abstract

The Phospholipase A2 Receptor 1 (PLA2R1) was first identified for its ability to bind some secreted PLA2s (sPLA2s). It belongs to the C-type lectin superfamily and it binds different types of proteins. It is likely a multifunctional protein that plays a role i) in inflammation and inflammatory diseases, ii) in cellular senescence, a mechanism participating in aging and age-related diseases including cancer, and iii) in membranous nephropathy (MN), a rare autoimmune kidney disease where PLA2R1 is the major autoantigen. To help study the role of PLA2R1 in these pathophysiological conditions, we have generated a versatile NeoR-hPLA2R1 conditional transgenic mice which will allow the specific expression of human PLA2R1 (hPLA2R1) in relevant organs and cells following Cre recombinase-driven excision of the NeoR-stop cassette flanked by LoxP sites. Proof-of-concept breeding of NeoR-hPLA2R1 mice with the ubiquitous adenoviral EIIa promoter-driven Cre mouse line resulted in the expected excision of the NeoR-stop cassette and the expression of hPLA2R1 in all tested tissues. These Tg-hPLA2R1 animals breed normally, with no reproduction or apparent growth defect. These models, especially the NeoR-hPLA2R1 conditional transgenic mouse line, will facilitate the future investigation of PLA2R1 functions in relevant pathophysiological contexts, including inflammatory diseases, age-related diseases and MN.

## Introduction

PLA2R1 was initially identified for its ability to bind some sPLA2s^1,2^. It belongs to the C-type lectin superfamily and it also binds collagen and various sugars^3,4^. The roles of PLA2R1 and functional consequence of PLA2R1 binding to sPLA2s, collagen and sugars are still not well understood.

sPLA2s catalyze the hydrolysis of phospholipids to generate lysophospholipids and fatty acids which are precursors of numerous pro- and anti-inflammatory lipid mediators. Binding of sPLA2s to PLA2R1 leads to inhibition of their enzymatic activity, their internalization and their degradation, suggesting a role of PLA2R1 in controlling sPLA2 function^5–9^. For example, inhibition of sPLA2s by PLA2R1 protects mice in a model of ovalbumin-induced lung inflammation^10,11^. However, other studies suggest that PLA2R1 may promote sPLA2 biological effects, especially for PLA2G1B, or may function independently of sPLA2s. For instance, PLA2R1-deficient mice appear more resistant to LPS-mediated endotoxic shock^12^ and more susceptible to cardiac rupture after myocardial infarction^13^, by mechanisms possibly linked to sPLA2s. *In vitro*, PLA2G1B may promote the proliferation of pancreatic cells^14^ while inducing the death of kidney podocytes via PLA2R1^15^. PLA2R1 may also play a role in cell adhesion via its collagen binding properties^2,3,16^.

Emerging results also suggest a role of PLA2R1 as a driver of cellular senescence contributing to progeria and tumor initiation processes in mice^17–19^. In cancer cells, PLA2R1 expression is generally decreased and its constitutive expression results in tumor growth inhibition while its reduction fosters cancer progression^19–21^. The underlying mechanisms of action of PLA2R1 seem to be independent of sPLA2 binding^22,23^.

Finally, PLA2R1 has been identified as the major autoantigen in membranous nephropathy (MN), a rare autoimmune kidney disease^24^. Indeed, autoantibodies against PLA2R1 were detected in about 70% of patients with MN. These autoantibodies form immune complexes with PLA2R1 at the surface of podocytes in the kidney glomerulus. The discovery of PLA2R1 autoantibodies has led to a rapid paradigm shift in diagnosis and monitoring of MN disease^25–27^. Although anti-PLA2R1 patients’ autoantibodies are likely involved in MN pathogenesis, the definitive proof that binding of these autoantibodies to PLA2R1 at the surface of podocytes can induce MN remains to be obtained, and this cannot be easily tested in wild-type (WT) mice. Indeed, in contrast to human podocytes, mouse podocytes do not express PLA2R1, thereby preventing the experimental evaluation of the role of the PLA2R1/anti-PLA2R1 interaction in a mouse model, as it was done for THSD7A, a second autoantigen of MN^28–30^.

The above non-exhaustive presentation of PLA2R1 functions emphasizes the importance of developing novel PLA2R1 overexpressing mouse models, which will complement studies with the existing *Pla2r1* KO mice^12^, to improve our understanding of the functions of PLA2R1 in inflammatory diseases, aging diseases, cancer and MN.

## Results and Discussion

The primary aim of this work was to generate and validate a transgenic mouse model allowing the conditional overexpression of hPLA2R1 driven by a Cre-recombinase approach, in order to subsequently generate various experimental models to study the different pathophysiological functions of PLA2R1 *in vivo*. We chose to express human PLA2R1 based on the different sPLA2 binding abilities reported for PLA2R1 orthologs^3,7,31^. Specifically, while several mouse sPLA2s are known to bind to mouse PLA2R1^31^, the binding properties of mouse and human sPLA2s to hPLA2R1 have only been partially elucidated and differ for at least PLA2G1B and PLA2G2A^3,7^. We also chose this strategy because of its high affinity for anti-PLA2R1 autoantibodies from MN patients^32–34^. However, this choice might present disadvantages as the binding properties of mouse sPLA2s to PLA2R1 may differ between PLA2R1 orthologs including hPLA2R1^3^ and because human anti-PLA2R1 autoantibodies are poor triggers of complement activation in mice, especially during the initial heterologous phase after passive transfer of human autoantibodies^30,32^. As the mouse *Pla2r1* will remain expressed in transgenic hPLA2R1 mice, it might also add a confounder effect that might complicate the interpretations of some data, but this could be circumvented by performing experiments in *Pla2r1* KO mice if necessary^12,19^.

**Figure 1:**
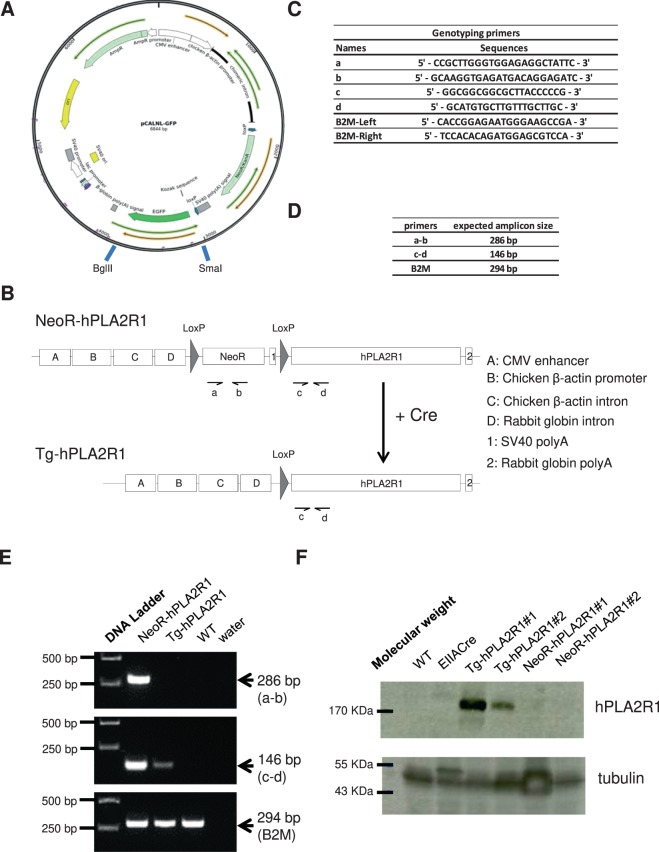
Strategy to generate NeoR-hPLA2R1 and Tg-hPLA2R1 mice. (**A**) Map of pCALNL-GFP backbone vector used for transgenesis. The pCALNL/hPLA2R1 vector was generated by replacing the GFP coding sequence by that of hPLA2R1. (**B**) Schematic representation of NeoR-hPLA2R1 and Tg-hPLA2R1 transgenic constructs prior to and after Cre excision of the LoxP-NeoRStop-LoxP sequence. Arrows show the position of genotyping primers. (**C**) Sequences of the primers used for genotyping NeoR-hPLA2R1 and Tg-hPLA2R1 transgenic mice. Beta-2-microglobulin (B2M) was used as an internal gDNA control. (**D**) Expected size of the respective amplicons obtained after PCR amplification with indicated primers. (**E**) Representative picture of a genotyping PCR run on an agarose gel and stained with SYBR-safe DNA dye. Amplicon bands and their respective sizes are indicated by arrows. (**F**) Protein lysates prepared from liver of 3-months old mice were analyzed by immunoblot. Tubulin was used as a loading control.

Construction of the transgenesis vector was achieved by inserting the 4.3 kbp hPLA2R1 full-length ORF cloned by PCR-amplification from a pLPCX/hPLA2R1 expression plasmid^19,22^ instead of GFP into the pCALNL/GFP vector, downstream of a LoxP-NeoR-Stop-LoxP sequence^35^ (Fig. 1A). After having checked hPLA2R1 integrity by sequencing, the linearized pCALNL/hPLA2R1 transgenesis vector was injected into the pronucleus of fertilized oocytes prior to their implantation into pseudopregnant mice. Identification of transgenic mice containing hPLA2R1 downstream of the LoxP-NeoR-Stop-LoxP sequence was subsequently performed by PCR on newborn pups (Fig. 1B–E). These transgenic mice were referred as NeoR-hPLA2R1 animals, which should allow the expression of hPLA2R1 after crossing them with mice expressing the Cre recombinase. This expression could be ubiquitous, tissue-specific and/or inducible pending of the tissue specificity and/or inducibility of the expressed Cre recombinase.

To assess the general functionality of NeoR-hPLA2R1 mice, we bred them with EIIa-Cre transgenic animals that express the Cre recombinase under the control of the adenoviral EIIa-promoter, an ubiquitous promoter^36^. By this means, we generated Tg-hPLA2R1 mice that should display an ubiquitous Cre-mediated excision of the LoxP-NeoR-Stop-LoxP sequence. Recombination of the LoxP-NeoR-Stop-LoxP cassette was verified by PCR analysis performed on DNA extracted from tail-tissue of 7 days old newborn mice (Fig. 1B–E) and by immunoblot for protein expression on liver of 3 months old mice (Fig. 1F). As expected, the hPLA2R1 transgene was defloxed and the protein was clearly expressed in liver tissue in Tg-hPLA2R1 but not WT animals.

**Figure 2:**
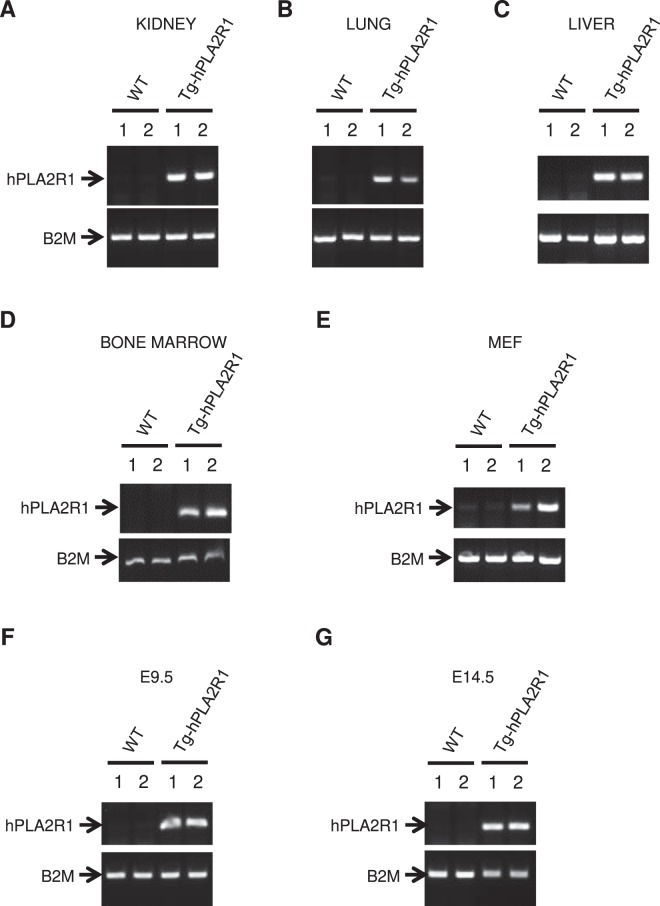
Tissue genotyping on mice, embryos and their derived cells. (**A**–**D**) PCR analysis of extracted DNA obtained from 6 months old Tg-hPLA2R1 and WT mouse tissues. Representative results of the PCR performed on kidney (**A**), lung (**B**), liver (**C**) and bone marrow (**D**) DNA. (**E**) Representative results of the PCR performed on DNA extraction from the second passage of E13.5dpc-isolated MEFs. (**F**,**G**) Representative results of PCR performed on DNA obtained from E9.5dpc whole embryo and from embryonic livers dissected at E14.5dpc. Two WT and two Tg-hPLA2R1 littermate samples (indicated as 1 and 2) were used in all experiments.

Tg-hPLA2R1 mice were amplified by breeding them with Wild Type (WT) mice to generate a littermate cohort (Table 1) and genotypes were verified on kidney, lung, liver and bone marrow DNA obtained from adult Tg-hPLA2R1 mice, in E9.5 days post coitum (dpc) whole embryos, E14.5dpc fetal livers and in Tg-hPLA2R1-derived mouse embryonic fibroblasts (MEFs) (Fig. 2).

**Table 1:** Progeny analysis and Mendelian repartition of WT × Tg-hPLA2R1 or WT x NeoR-hPLA2R1 breeding.

Breeding Schemes	Gender of Transgene carrier	Number of cross	Number of litter	Number of mice	Mice per litter	expected genotypes	Number/total analyzed	Calculated ratio (% to total)	expected Mendelian ratio (%)
Tg-hPLA2R1 × WT	♂	16	31	205	6.61	WT	123/205	60.00	50.00
						Tg-hPLA2R1	82/205	40.00	50.00
	♀	14	33	236	7.15	WT	129/236	54.66	50.00
						Tg-hPLA2R1	107/236	45.34	50.00
NeoR-hPLA2R1 × WT	♂	4	7	41	5.86	WT	21/41	48.78	50.00
						NeoR-hPLA2R1	20/41	51.22	50.00
	♀	4	9	59	6.56	WT	27/51	50.85	50.00
						NeoR-hPLA2R1	24/51	49.15	50.00

**Figure 3:**
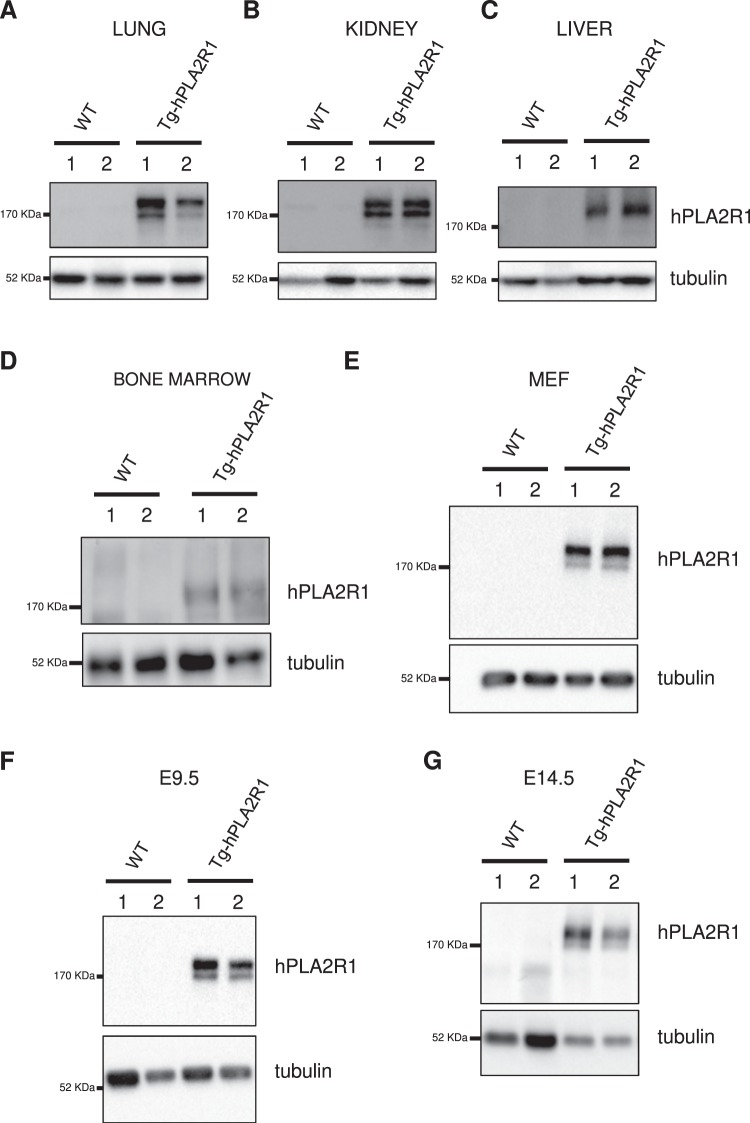
Expression of the hPLA2R1 protein in adult tissues, embryos and MEFs. (**A**–**D**) Protein lysates of lung (**A**), kidney (**B**), liver (**C**) and bone marrow (**D**) were prepared from 6 months old mice and analyzed by Western-blot using anti-PLA2R1 and anti-tubulin antibodies. (**E**–**G**) Expression of hPLA2R1 protein in E13.5-dpc derived MEFs (**E**), E9.5dpc whole embryo (**F**) and E14.5dpc fetal-liver (**G**) are shown. Immunoblots are represented for 2 WT and 2 Tg-hPLA2R1 littermate samples (indicated as 1 and 2) in all experiments.

We next investigated whether Tg-hPLA2R1 mice expressed the hPLA2R1 protein in different tissues. To that end, we performed immunoblot experiments from lung, kidney, liver and bone marrow protein extracts and confirmed that those tissues expressed the hPLA2R1 protein in Tg-hPLA2R1 mice compared to WT control littermates (Fig. 3A–D). Consistently, hPLA2R1 expression was detected in Tg-hPLA2R1 MEFs, E9.5dpc embryos and E14.5dpc fetal livers (Fig. 3E–G). These results demonstrate that hPLA2R1 is expressed in Tg-hPLA2R1 mice. Of note, hPLA2R1 was expressed as one or two bands of different ratios depending on tissue, which might be due to tissue-specific differences in PLA2R1 glycosylation and/or degradation.

**Figure 4:**
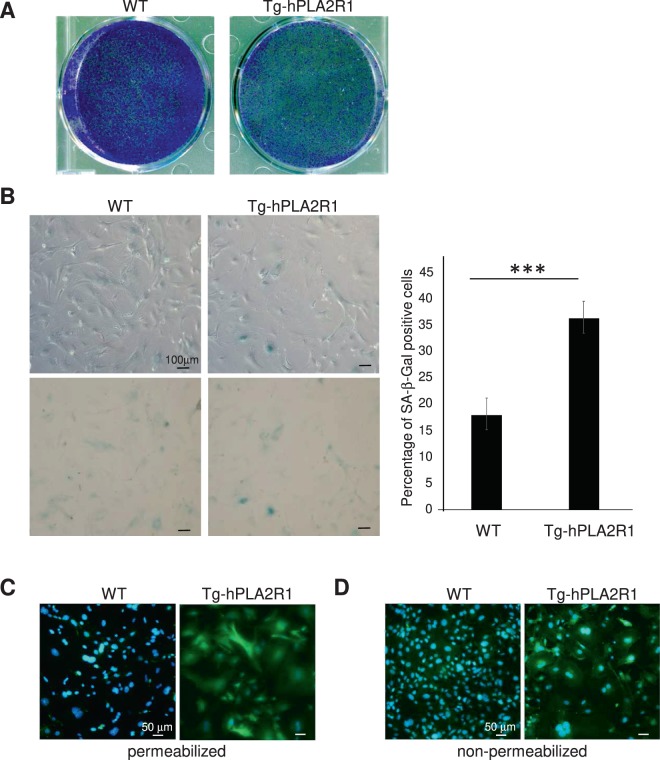
Tg-hPLA2R1 derived MEFs senesce prematurely. (**A)** Indicated MEFs were seeded at the same cell density and were PFA-fixed and stained by crystal violet 9 days later (**A**) or analyzed for SA-β-Gal activity 7 days later (**B**). Representative photographs and percentage of stained cells (means +/− SD, and p-value using student t-test, ***P < 0.005) are shown. (**C**) Cells were methanol fixed (permeabilized conditions), or (**D**) fixed using PFA (non-permeabilized conditions), and (**C**,**D**) IF against PLA2R1 was performed. Nuclear counterstaining was performed using Hoechst dye.

To ascertain whether the expressed hPLA2R1 protein was functional, we used MEFs derived from WT and Tg-hPLA2R1 embryos and cultured them to examine the cellular senescence phenotype, a phenotype well-known to be regulated by PLA2R1^17–19^. hPLA2R1 was clearly functional as it induced premature senescence of MEFs according to the decreased number of cells (Fig. 4A) and the increased proportion of senescence-associated-β-Galactosidase activity (SA-β-Gal)-positive cells (Fig. 4B). Immunofluorescence staining of hPLA2R1 in MEFs derived from Tg-hPLA2R1 embryos under non-permeabilized versus permeabilized conditions confirmed expression of hPLA2R1 at the plasma membrane and intracellularly (Fig. 4C,D).

**Figure 5:**
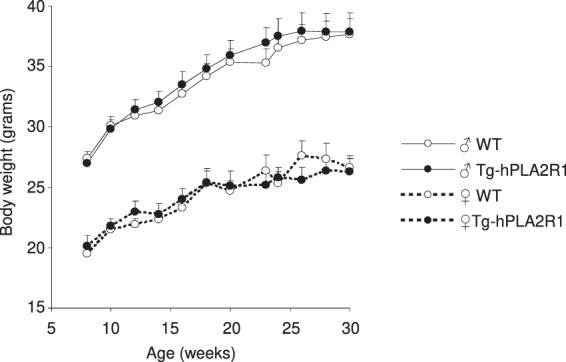
Body weight curves of Tg-hPLA2R1 and WT littermate mice. WT (males n = 10 and females n = 8) and Tg-hPLA2R1 (males n = 8 and females n = 5) animals were weighted every 2–3 weeks from 6 weeks to 30 weeks of age. Curves represented the mean weight ± SEM. Sex and genotype are as indicated.

Having validated that Tg-hPLA2R1 mice expressed hPLA2R1 during embryogenesis and in adult tissues, we next sought to address whether the expression of hPLA2R1 impacted the embryonic development and fertility of transgenic animals. Statistical analysis of the number of animals obtained from WT × Tg-hPLA2R1 breeding confirmed that the Mendelian distribution of males and females WT and Tg-hPLA2R1 newborn mice was not significantly different, highlighting the absence of an embryonic lethality linked to the expression of hPLA2R1 (Table 1). To the same extent, comparative analysis of the mean number of pups *per* litter obtained from NeoR-hPLA2R1 or Tg-hPLA2R1 animals bred with WT mice, revealed no defects in the fertility of Tg-hPLA2R1 recombined males and females (Table 1). Finally, analysis of the weight curves of sex matched WT and Tg-hPLA2R1 littermates over 30 weeks revealed no significant difference, indicating that the expression of hPLA2R1 did not influence the growth of young Tg-hPLA2R1 adult mice (Fig. 5). Considering the previously described major effects of PLA2R1 on senescence^17–19^ that we confirmed here using Tg-hPLA2R1 derived MEFs (Fig. 4), we would have expected to observe premature aging of these mice. As we conducted this initial analysis with 6 months old mice, older mice and/or pro-aging challenges might be required to observe an accelerated aging phenotype in hPLA2R1-Tg mice.

Taken together, we report the generation and validation of two new mouse models that will facilitate the study of PLA2R1 function *in vivo*. While the NeoR-hPLA2R1 mouse model should provide a tissue-specific and/or inducible expression of hPLA2R1 upon Cre-mediated excision, for example after a podocyte-specific expression of the Cre^37,38^ to address questions related to MN^24,32,39–41^, our analysis of Tg-hPLA2R1 animals confirms that the ubiquitously induced expression of hPLA2R1 is viable and does not affect the breeding potential of Tg-hPLA2R1 male and female mice. Both models are therefore powerful tools that should help decipher and investigate the *in vivo* contribution of PLA2R1 to inflammation, aging, cancer and MN, in which hPLA2R1 likely plays a critical role.

## Methods

### Generation of human PLA2R1 transgenic mice and genotyping

All animal maintenance and experimental protocols were performed in accordance with the animal care guidelines of the European Union and French laws and were approved by the CECCAPP local Animal Ethic institutional Committee. Mice were grown and maintain for all the experiments in specific pathogen-free animal house.

hPLA2R1 ORF was obtained from a previously generated pLPCX-hPLA2R1 vector^19,22^. Briefly, hPLA2R1 was amplified with a Phusion High-Fidelity DNA polymerase kit (New England BioLabs) using the following forward and reverse primers: PLA2R1-SmaI, 5′-ATCCCGGGATGCTGCTGTCGCCGTCGCTGCTGC-3′ and PLA2R1-BglII, 5′-GAAGATCTTATTGGTCACTCTTCTCAAG-3′. The hPLA2R1 SmaI-BglII digested PCR fragment was then cloned into a pCALNL-GFP vector following the excision of the SmaI-GFP-BglII sequence^35^. Lack of mutation was validated by sequencing the cloned hPLA2R1 (Beckman Coulter Genomics).

The pCALNL-hPLA2R1 vector was digested by Sfi1 and Pvu1 and the linearized fragment of 8.7 kbp was purified and microinjected into the pronuclei of fertilized single cell mouse embryos (mouse strain FVBxB6D2F1). The eggs were then surgically transferred to the oviducts of time-mated pseudo-pregnant foster mothers, generated by mating females with vasectomized males. Founder animals were then backcrossed with C57BL/6 mice to validate the germline transmission to offspring and to generate F1 NeoR-hPLA2R1 mice. Tg-hPLA2R1 mice were generated by crossing F3 NeoR-hPLA2R1 mice with C57BL/6 *EIIa-Cre* mice^36^. Tg-hPLA2R1 mice used in this study were obtained by at least 2 backcrosses with C57BL/6 mice.

For genotyping, genomic DNA was extracted from 2 to 4-mm tail biopsies of 8-10 days old mice as previously described^19^. Thirty-five cycles of PCR were performed using the primers indicated in Fig. 1C using GoTaq G2 Flexi DNA polymerase kit (Promega). PCR products and molecular size marker (Generuler 1 kbp DNA ladder, Life Technologies) were run on 2% agarose gels and stained using the SYBR-safe DNA dye (Life Technologies).

### Tissue recombination analysis

Liver, lung, kidney and bone marrow were quickly removed and frozen in liquid nitrogen following mouse cervical dislocation. Tissues were stored at −80 °C prior to cryogenic grinding (Cryotec) and subsequent DNA extraction. Genotyping was performed on genomic DNA extracted from WT, NeoR/PLA2R1 and Tg-hPLA2R1 mice. Sequences of the genotyping primers are indicated in Fig. 1C. PCR products were run on 2% agarose gels and stained using SYBR-safe DNA dye (Thermofisher).

### Western blot analysis

Proteins were extracted with a RIPA buffer for cryoground liver or with Laemmli buffer for cryoground lung and kidney. Proteins were quantified using the spectroscopy method at 280 nm (preceded by Bradford staining for RIPA buffer). Fifty μg of proteins were separated by SDS-PAGE under reducing conditions and transferred onto nitrocellulose membranes. Membranes were blocked with 5% milk in TBS-Tween (0.05%) prior to incubation with a mouse monoclonal anti-PLA2R1 antibody (1/1000, AMAB90772, Atlas Antibodies) or tubulin loading control antibody (1/5000, T6199, Sigma). Peroxidase-coupled donkey anti-mouse IgG antibody (715-035-150, Jackson ImmunoResearch Europe Ltd) was added before visualization using an enhanced chemiluminescence kit (GE healthcare).

### Production of MEFs and senescence analysis

Mouse embryonic fibroblasts (MEFs) were isolated under sterile conditions, from E13.5dpc embryos obtained from WT mice bred with Tg-hPLA2R1 animals. Following embryo decapitation and fetal liver removal, the fibroblasts were dissociated and cultured in DMEM Glutamax (Gibco), with 15% fetal bovine serum (Biowest), 10 μM non-essential amino acids and penicillin/streptomycin (NEAA/PS, Gibco).

For senescence analysis by colony assay, cells were washed with PBS, fixed for 15 min in 3.7% formaldehyde, stained with 0.5% crystal violet solution overnight and finally rinsed in water. For SA‐β‐gal assay, cells were washed twice with PBS and fixed for 10 min in 0.5% glutaraldehyde. Cells were then rinsed twice in PBS and incubated at 37 °C overnight in SA‐β‐Gal solution as previously described^17–19^.

### Immunofluorescence

Cells were fixed for 10 min in 3.7% formaldehyde or in cold methanol to permeabilize the membranes, then washed with PBS-Tween (0.05%). Blocking of non-specific binding sites was achieved using 20% fetal bovine serum in PBS-Tween 0.01%. Staining was performed using anti-PLA2R1 Atlas antibody (AMAb90772), diluted to 1/250 in 20% fetal bovine serum and incubated overnight at 4 °C in a humidified chamber. After washes with PBS-Tween 0.05%, cells were incubated with a secondary anti mouse Alexa Fluor488 antibody (A21200, Life technologies) for 1 h at room temperature in a dark humidified chamber. After 10 min of staining with Hoechst 1/1000 in PBS, the cells were rinsed in PBS-Tween 0.05% and the slides were protected with coverslips secured with mounting medium (Southern Biotech Fluoromount G). Pictures were obtained under a Nikon microscope using CCD camera.

## Supplementary information

Supplementary information.

## Data Availability

All data generated or analyzed during this study are included in this published article.
