# Supplementary material for: Generation of a conditional transgenic mouse model expressing human Phospholipase A2 Receptor 1
Source: Sci Rep. 2020 May 18;10:8190. doi: 10.1038/s41598-020-64863-y (PMC7235081; doi:10.1038/s41598-020-64863-y)

Supplemental information – uncropped gel and western-blot

Generation of a conditional transgenic mouse model expressing human Phospholipase A2 Receptor 1

Sara Jaber, Delphine Goehrig, Philippe Bertolino, Amélie Massemin, Franck Bihl, Joëlle Chabry, Gérard Lambeau, David Vindrieux, David Bernard

To generate the Figure 1E

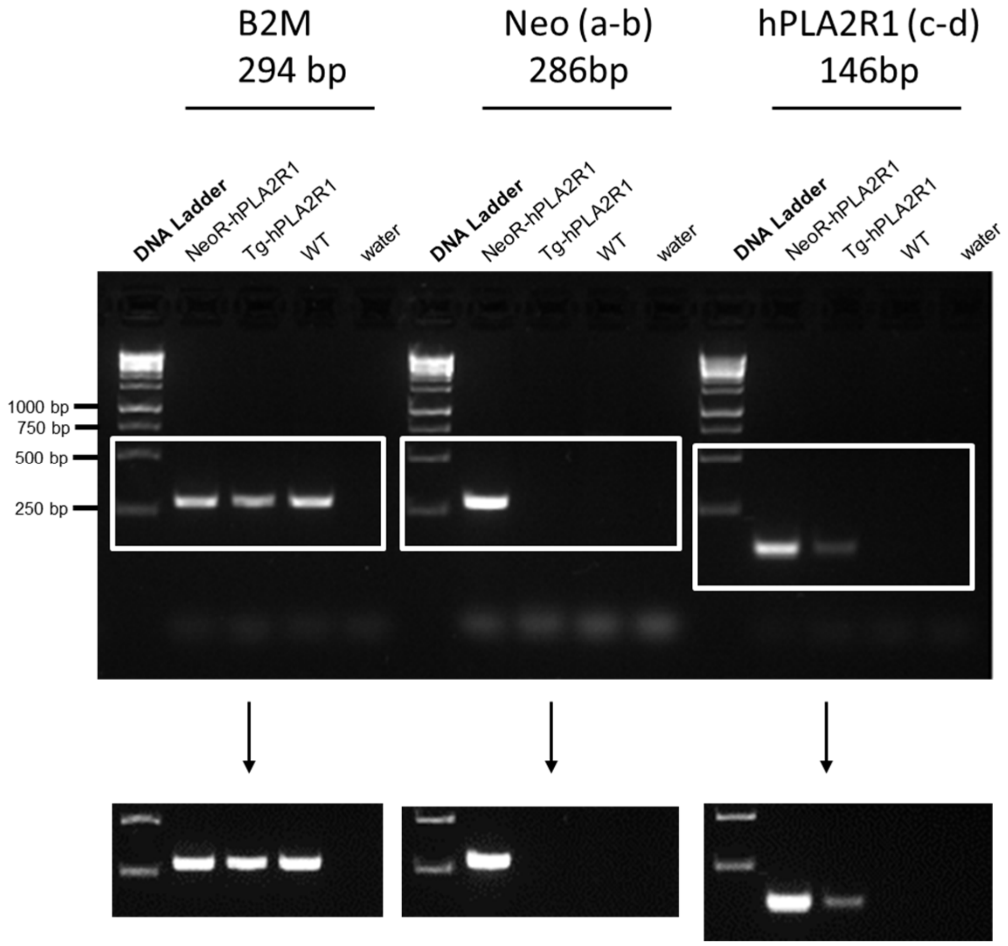

To generate the Figure 1F

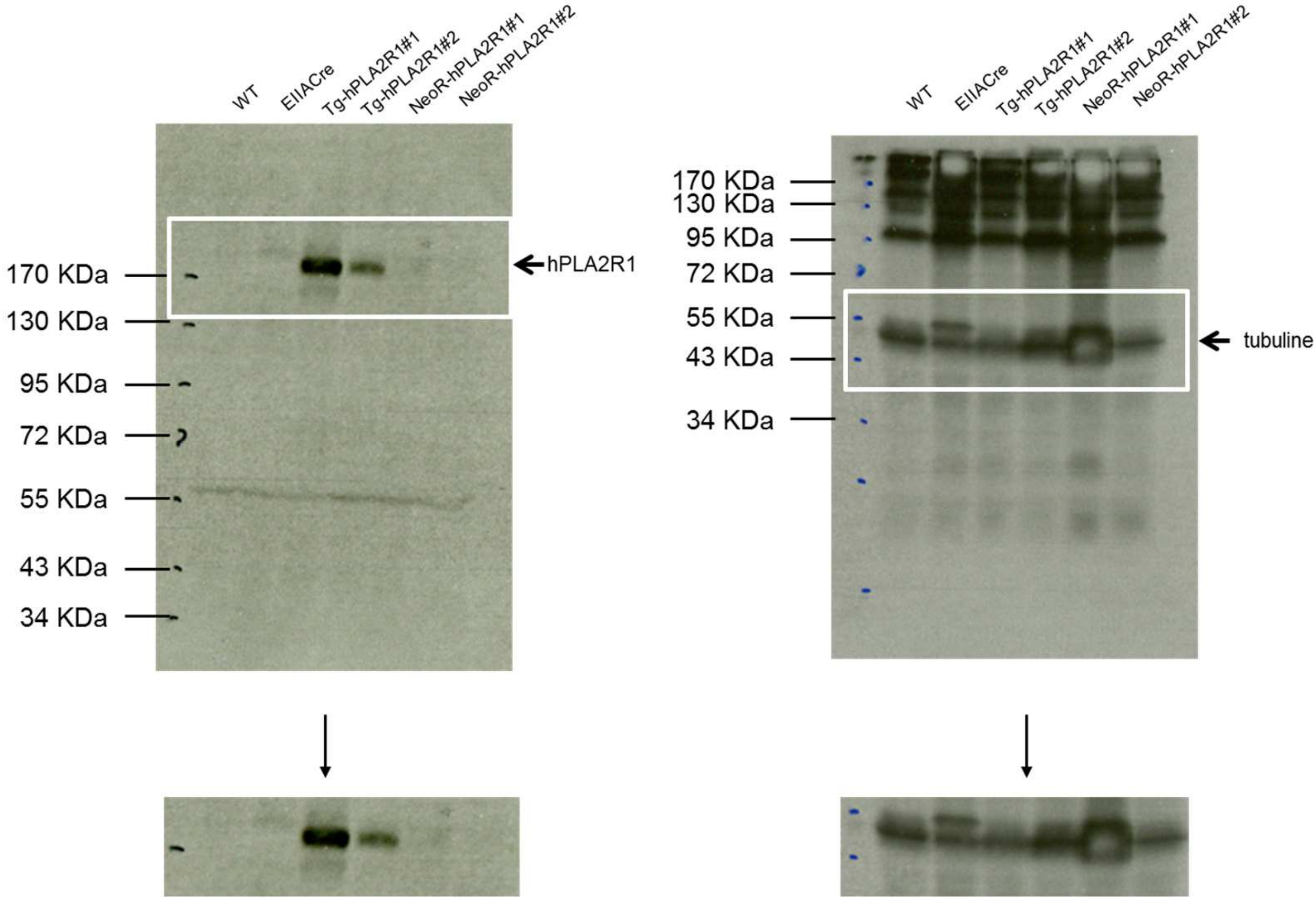

To generate the Figure 2A-C

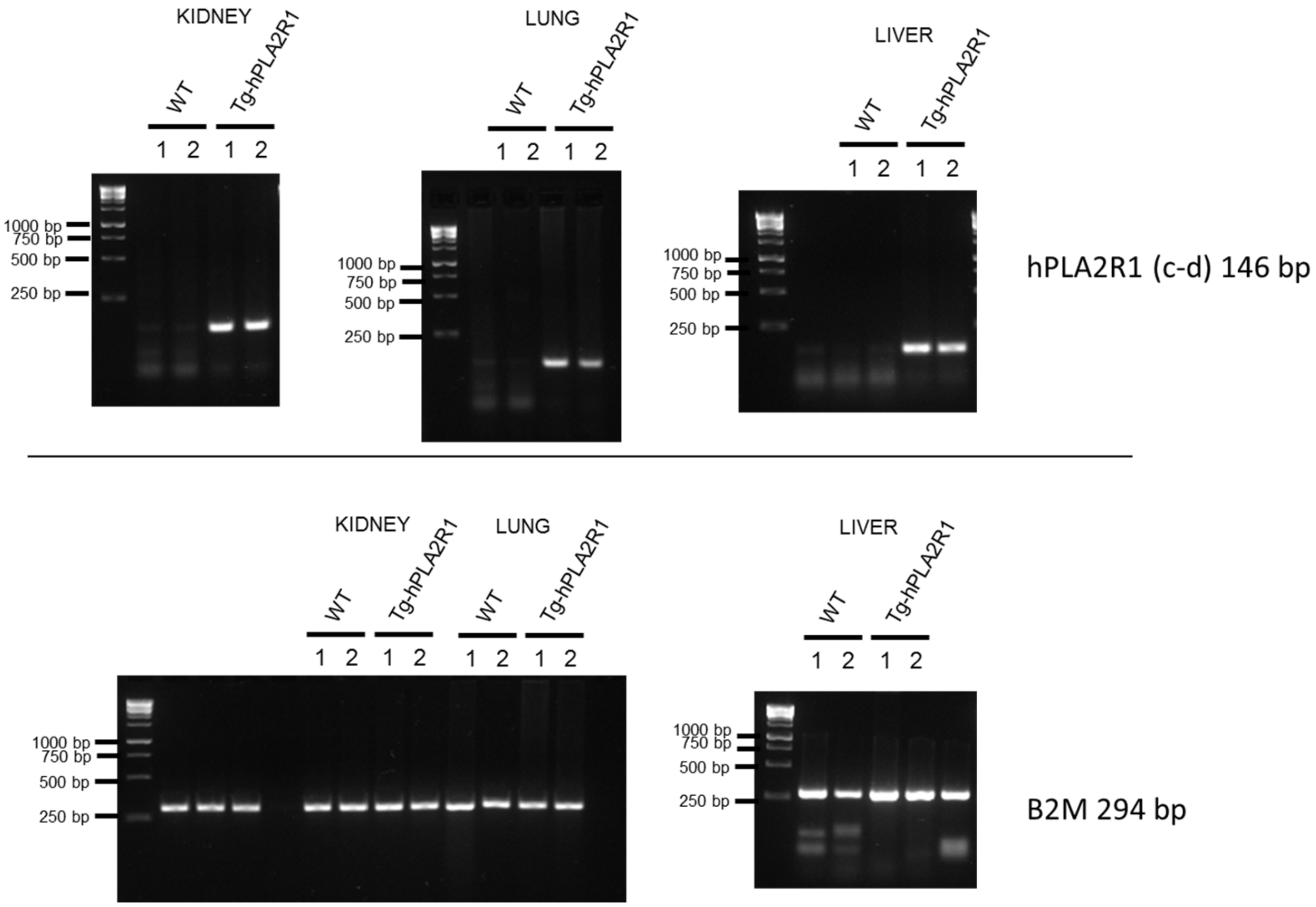

To generate the Figure 2D

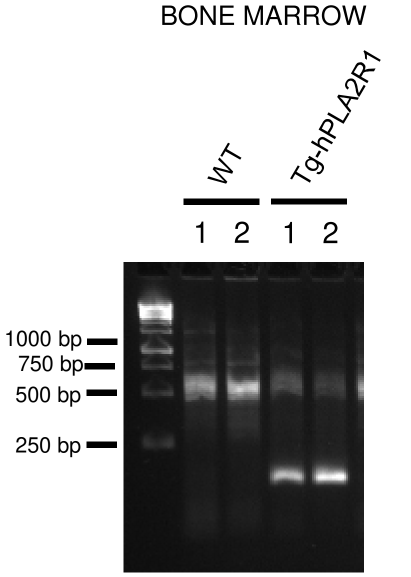

hPLA2R1 (c-d) 146 bp

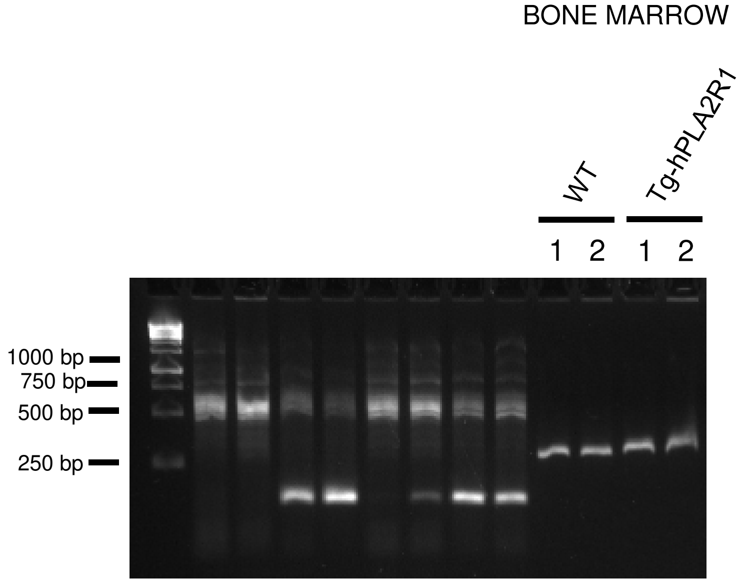

B2M 294 bp

To generate the Figure 2E-G

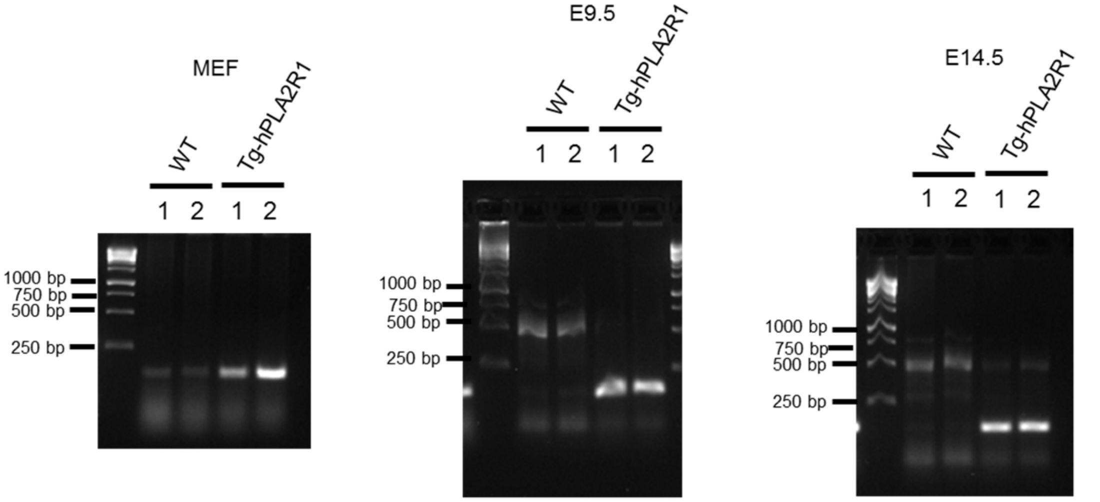

hPLA2R1 (c-d) 146 bp

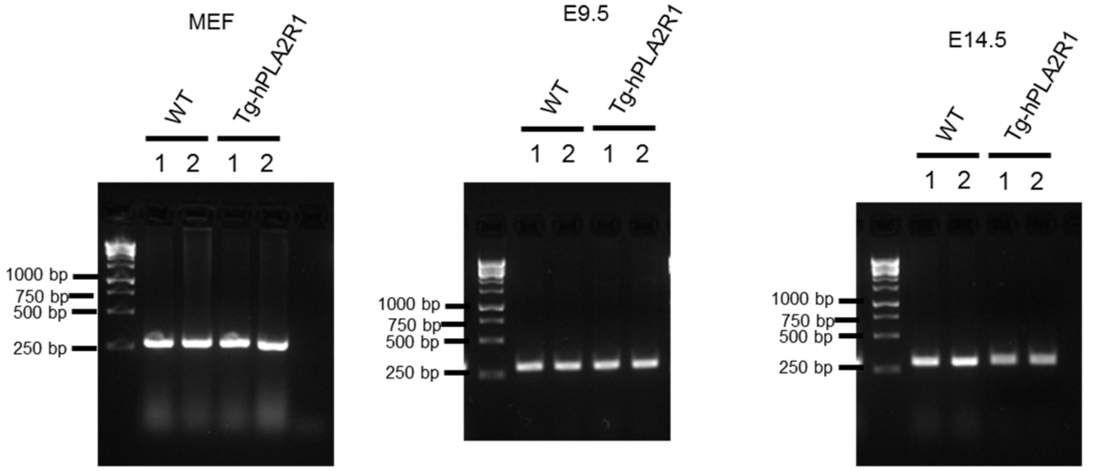

B2M 294 bp

To generate the Figure 3A-B

lung

kidney

PLA2R1

Tubulin

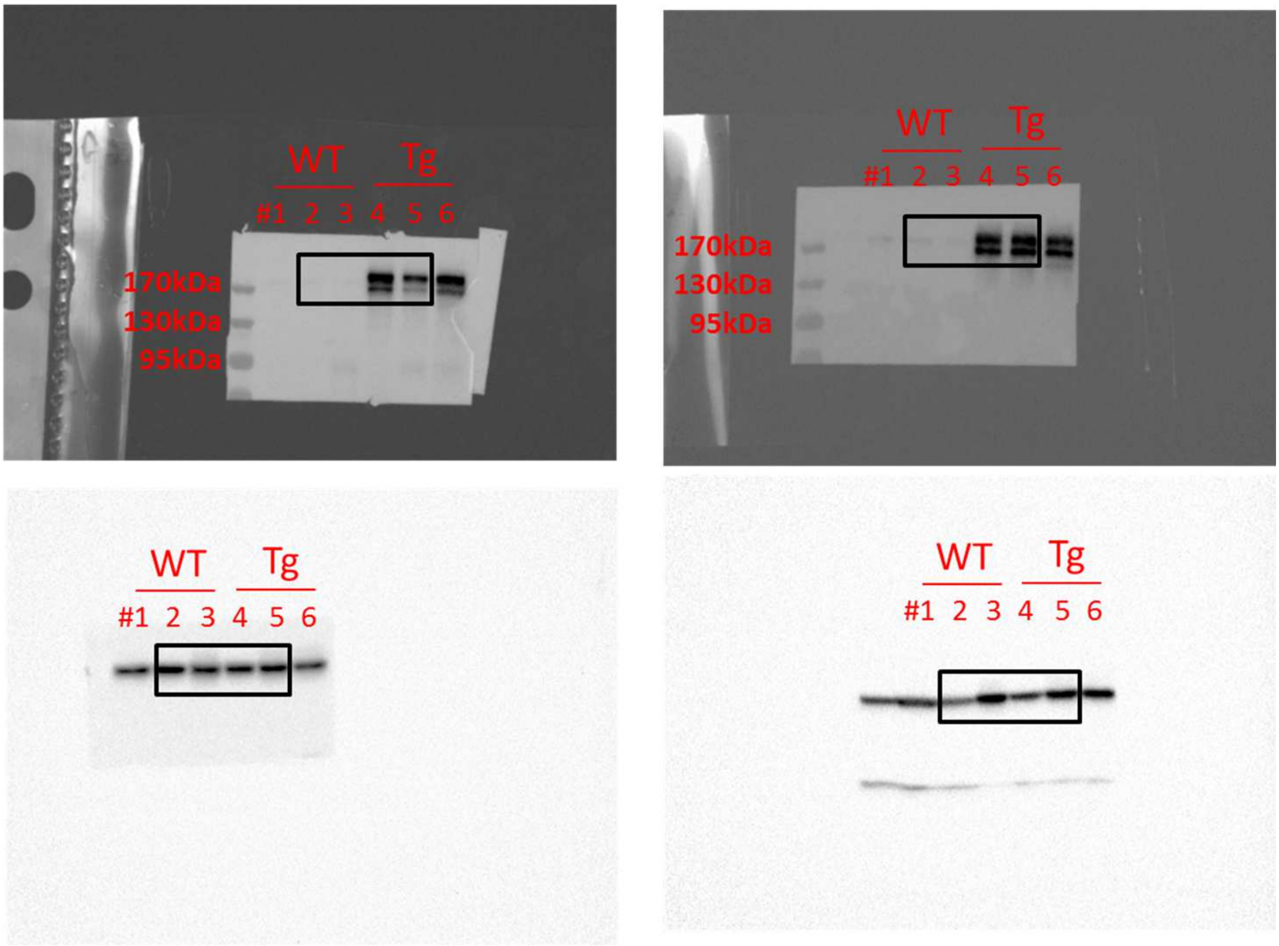

To generate the Figure 3C and 3E

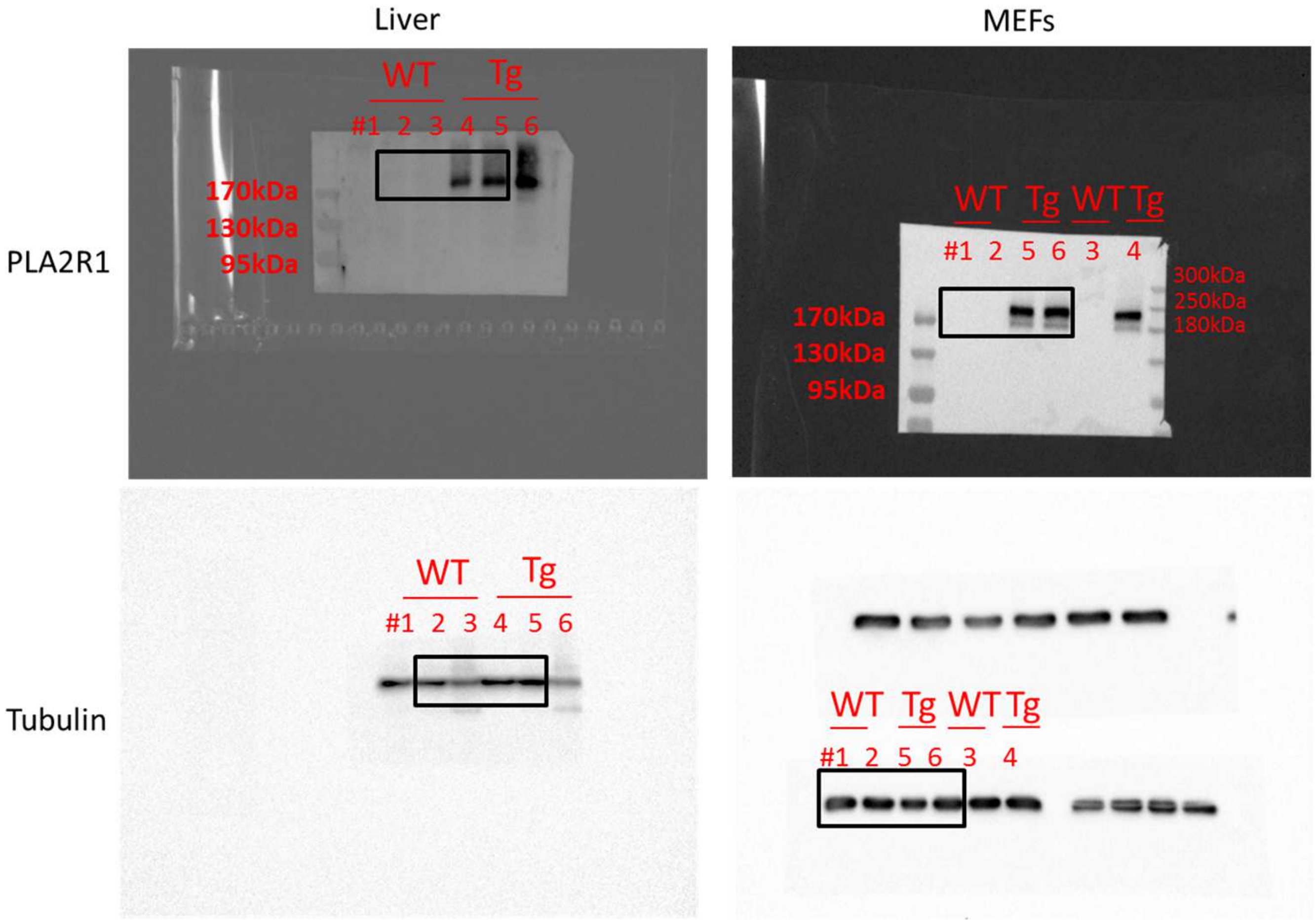

To generate the Figure 3D

PLA2R1

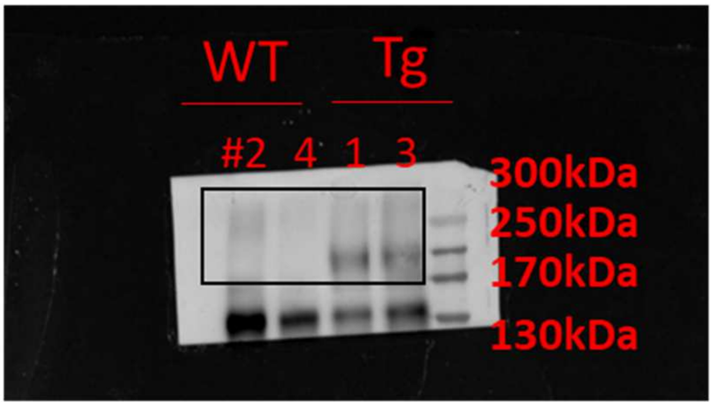

Tubulin

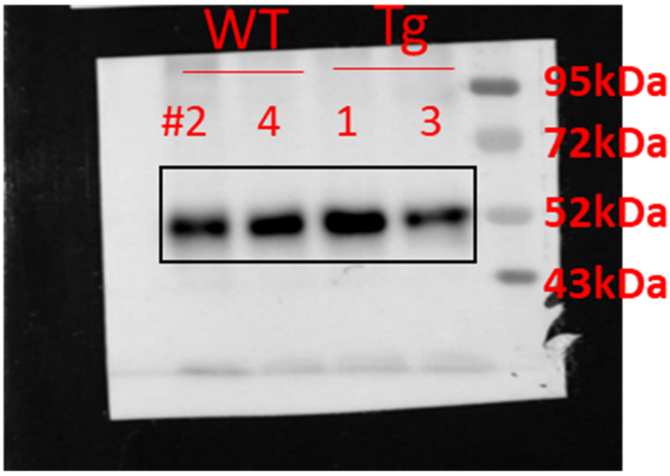

To generate the Figure 3F-G

E9.5

E14.5

PLA2R1

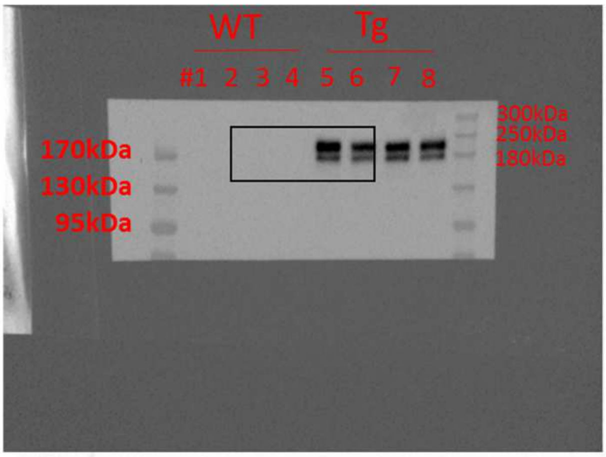

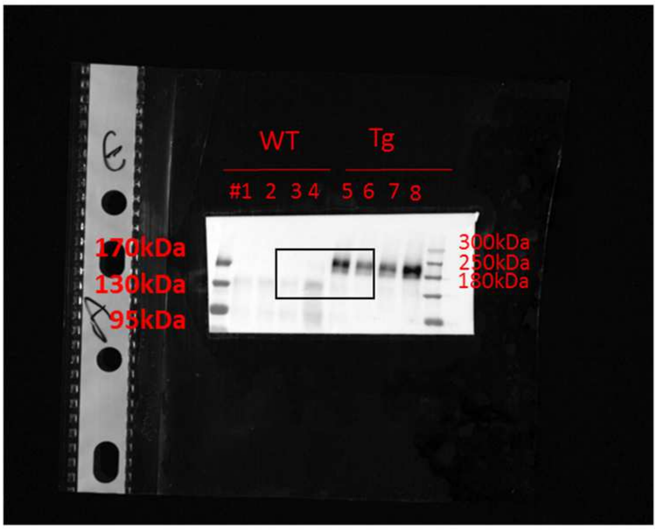

Tubulin

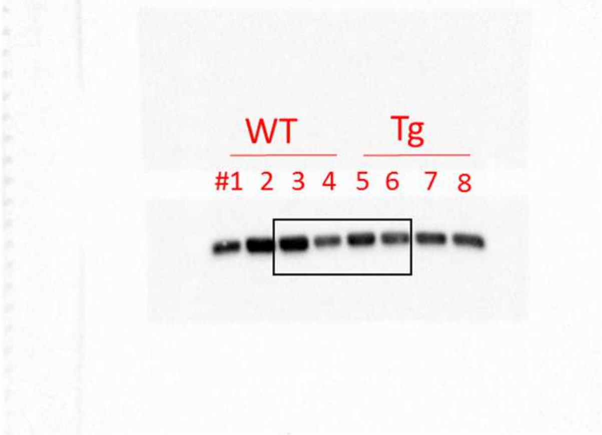

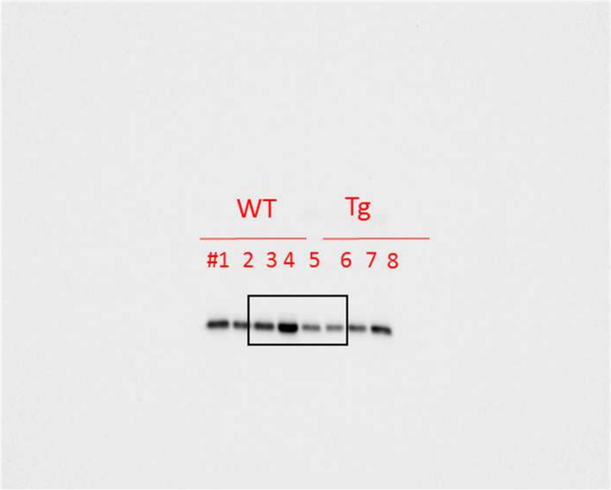

Supplement: Supplementary file 1 — Supplementary information. [file 41598_2020_64863_MOESM1_ESM.pdf]
